# Prevalence-Dependent Costs of Parasite Virulence

**DOI:** 10.1371/journal.pbio.0030262

**Published:** 2005-07-19

**Authors:** Stephanie Bedhomme, Philip Agnew, Yuri Vital, Christine Sidobre, Yannis Michalakis

**Affiliations:** **1**Department of Biology, Queen's University, Kingston, Ontario, Canada,; **2**Génétique et Evolution des Maladies Infectieuses, Montpellier, France; University of BaselSwitzerland

## Abstract

Costs of parasitism are commonly measured by comparing the performance of infected groups of individuals to that of uninfected control groups. This measure potentially underestimates the cost of parasitism because it ignores indirect costs, which may result from the modification of the competitiveness of the hosts by the parasite. In this context, we used the host-parasite system consisting of the yellow fever mosquito *Aedes aegypti* and the microsporidian parasite *Vavraia culicis* to address this question: Do infected individuals exert a more or less intense intraspecific competition than uninfected individuals? Our experimental results show that, indeed, infected hosts incur a direct cost of parasitism: It takes them longer to become adults than uninfected individuals. They also incur an indirect cost, however, which is actually larger than the direct cost: When grown in competition with uninfected individuals they develop even slower. The consequence of this modification of competitiveness is that, in our system, the cost of parasitism is underestimated by the traditional measure. Moreover, because the indirect cost depends on the frequency of interactions between infected and uninfected individuals, our results suggest that the real cost of parasitism, i.e., virulence, is negatively correlated with the prevalence of the parasite. This link between prevalence and virulence may have dynamical consequences, such as reducing the invasion threshold of the parasite, and evolutionary consequences, such as creating a selection pressure maintaining the host's constitutive resistance to the parasite.

## Abstract 2

The outcome of competition between mosquito larvae infected with a parasite depends on the infection status of the individual concerned and on that of its competitor, with implications for measuring virulence and for host-parasite dynamics.

## Introduction

The presence of parasitism creates heterogeneity in host populations: Parasitised hosts may have different behaviour [1], different food requirements [2], different feeding rates [3], or different sensitivity to stress, such as pollution [4]. This heterogeneity can modify intraspecific competition among individuals within host populations. There are three types of competitive interaction to consider: Among uninfected hosts, among infected hosts, or between uninfected and infected hosts. If these interactions differed in their effects on host life-history traits, the presence of parasitism would induce not only a direct cost of infection but also indirect costs through the modification of intraspecific competitiveness. If infected hosts differ in competitive strength from uninfected hosts, the outcome of competitive interactions will depend on which individuals are involved, and thus indirect costs will depend on the parasite's prevalence.

These potential indirect effects of parasitism are especially important within the context of measuring the costs of parasitism. Indeed, what is typically measured is the direct cost only; normally, this is done by comparing the performance of individuals coming from either infected or uninfected populations, and the indirect costs resulting from the modification of the competitive ability of infected individuals are not taken into account.

We used the system involving the yellow fever mosquito *Aedes aegypti* and the microsporidian parasite *Vavraia culicis* to investigate whether infected hosts exert more or less intense intraspecific competition than uninfected hosts. That is to say, we investigated whether the parasitism modifies the host's intraspecific competitiveness. We addressed this issue in our experiment by measuring both direct and indirect costs, by comparing intraspecific competition between and among parasitised and unparasitised individuals. More specifically, two individuals, which were infected or not, competed for resources in standard growing vials. This resulted in four treatments, according to the infection status of competing larvae, which were: (i) infected versus infected (+/+), (ii) infected versus control (+/−), (iii) control versus infected (−/+), and (iv) control versus control (−/−), where the first term refers to the focal individual and the second to its competitor. To take into account possible interactions with the amount of resources available, we considered two food regimes, 100% and 50% of a standard regime detailed in the Materials and Methods section. We measured the direct and indirect effects of parasitism through the modifications it induced on the probability to emerge, i.e., the probability to reach the adult stage, and on developmental time of the focal individual as a function of the infection status of the focal individual and its competitor.

## Results

The probability of both individuals emerging when receiving a 100% diet was high and similar for the “−/−” and “+/+” treatments (89.6% and 86.3%, respectively). This probability remained high when receiving a 50% regime for the “−/−” treatment (94.4%) but was lower for the “+/+” treatment (67.8%) and caused a significant food-by-treatment interaction (chi-square = 8.823, degrees of freedom [d.f.] = 1, *p* = 0.003). The frequency of pre-emergence mortality observed in the “+/−” treatments however was not different from that which would be expected on the combined mortality of infected and uninfected individuals estimated from the “+/+” and “−/−” treatments (100% regime, chi-square = 1.458, d.f. = 1, *p* = 0.227; 50% regime, chi-square = 0.635, d.f. = 1, *p* = 0.235). Furthermore, of the 19 cases where only one individual emerged from a “+/−” treatment receiving the 50% regime, 11 were uninfected individuals and eight were infected individuals, showing that pre-emergence mortality in “+/−” treatments was not strongly biased towards the infected individual. Thus, the 50% regime decreased the overall probability of an infected individual emerging, but there was no evidence that being in competition with an uninfected individual amplified this effect.

We found significant effects for the effect of food on developmental time (Table 1): Individuals with a 100% regime developed faster. A significant effect of the “infection status” was found for developmental time (Figure 1): Infected individuals had a longer developmental time. The effect of infection is also illustrated by considering the status of the first individual to emerge in “+/−” vials. Infected individuals emerged first only in 22.5% cases (Fisher's exact test *p* < 0.001). Controlling for gender composition showed that the infection effect is even stronger: In vials with two males, the infected individual emerged first in 19.6% of the cases (Fisher's exact test *p* < 0.001), while in vials with two females, the infected individual emerged first in 10.1% of the cases (Fisher's exact test *p* < 0.001). Moreover, as Figure 1 shows, individuals having an infected competitor (i.e., “−/+” and “+/+” individuals) had a shorter developmental time than individuals having an uninfected competitor (i.e., “+/−” and “−/−” individuals). The subsequent analyses allowed us to verify this influence of the nature of the competitor: “+/+” individuals had a shorter developmental time (*p* = 0.019) than “+/−” individuals, and “−/+” individuals had a shorter developmental time (*p* < 0.001) than “−/−” individuals. Sex status had a significant effect corresponding to known differences between male and female mosquitoes: Males had a shorter developmental time than females. However, the interaction between sex status and infection status was not significant.

**Figure 1:**
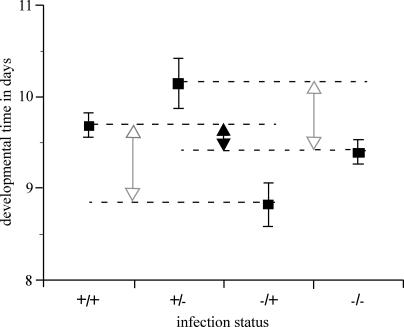
Developmental Time in Days and 95% Confidence Intervals for the Four Categories of Infection Status In the infection status category denominations (e.g., +/+, +/−, −/+, and −/−), the first symbol indicates the infection status of the focal individual, and the second indicates that of its competitor. The filled black arrow represents the commonly measured direct costs of parasitism, and the open grey arrows represent the indirect costs through the modification of intraspecific competitiveness by parasitism (see text).

**Table 1:**
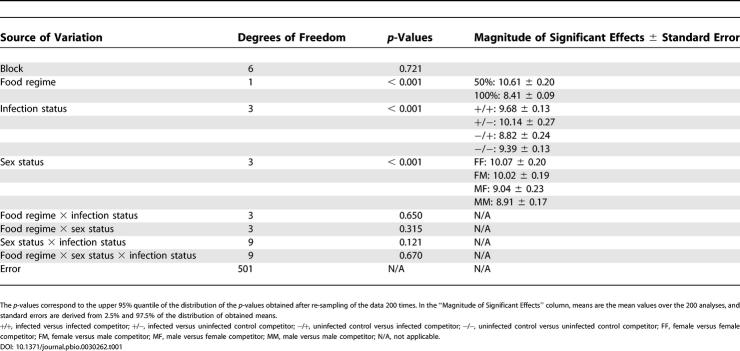
ANOVAS for the Effects of Food Regime, Sex Status, and Infection Status on Developmental Time

## Discussion

Our data indicate that infection causes an increase in developmental time of the host, which, when combined with lower larval food availability, decreases the probability of infected individuals surviving to emerge as adults. Moreover, we show that parasitism modifies intraspecific competitiveness for the host. Indeed, individuals grown with an infected individual had a shorter developmental time. This suggests that the intraspecific competition exerted by infected individuals is less intense than for uninfected individuals.

An increase in developmental time is costly for hosts not only because it lengthens generation time [5], but also because the rate of pre-adult mortality due to *V. culicis* increases with time [6].

For male mosquitoes, an increase in developmental time is likely to entail reduced reproductive success. This is because the development of larval populations often begins synchronously following the immersion of eggs by water and because adult females become refractory to copulation after being mated once. Thus, in male-male competition for females, slower larval development will be associated with reduced access to mating.

The less intense competition exerted by infected host individuals could be explained by a reduced feeding rate due to a less intense competitiveness for food. They could also show less physical activity and thus generate less stress by reduced contact with their competitors.

Our results could be interpreted as a simple variation in the expression of the costs of parasitism depending on environmental conditions, as has been shown for many other host-parasite systems [6–9]. However, in the present experiment, the variation in the expression of virulence is due to the presence of the parasite itself in other individuals of the host population and, therefore, implies feedbacks in dynamic and evolutionary processes, as discussed below.

Less intense competitiveness of infected individuals has already been reported in host-parasitoid interactions: Sisterson and Averill [10] showed that larvae of *Acrobasis vaccinii* (Lepidoptera), which are parasitised by the parasitoid *Phanerotoma franklini* (Hymenoptera), defended their resources better against parasitised rather than against unparasitised hosts. Other experimental studies have shown that parasitoidism may considerably modify the effects of intraspecific competition among hosts [10–15], and theoretical studies have suggested that it can be an important factor in their population dynamics [16–18]. However, the consequences of a modification in the intensity of intraspecific competition by parasitism on parasite virulence and its evolutionary consequences were not explored.

### Consequences for Measures of Virulence

The intensity of intraspecific competition was modified by parasitism in our system. This implies that the costs of parasitism are not completely captured by comparing individuals from a completely infected population to individuals of a completely uninfected population. If we concentrate on our results for developmental time, this kind of classical measurement of virulence is symbolised by the filled black arrow in Figure 1. This comparison between infected and uninfected populations underestimates the full cost of parasitism because it ignores the cost induced by the modifications of intraspecific competitiveness, represented by the open grey arrows in Figure 1. A more realistic evaluation of the full cost of infection should incorporate all types of costs, the relative weight of each type depending on the frequency of each type of competitive interaction in the population. In populations with a low prevalence of parasitism, infected individuals interact mainly with uninfected competitors. This type of competitive interaction is the most costly for infected hosts. Thus, the virulence expressed in populations with a low prevalence of infection may be high. In contrast, where the prevalence of infection is high, infected individuals mainly interact with other infected individuals, and thus will not pay the indirect cost of parasitism. Virulence may thus be negatively correlated with parasite prevalence. However, we used here a minimalist representation of prevalence, and at least two other phenomena could influence the shape of the relationship between prevalence and virulence. The first is that infected individuals could aggregate to reduce the fitness cost of infection by reducing their contact with uninfected individuals. The second is that high-prevalence conditions favor high parasite burden and multiple infections. High parasite burden increases the negative effects of parasites. Multiple infection, on the other hand, may either increase [19] or decrease [20–22] parasite virulence, depending on how various factors interact to determine parasitic virulence. The slope of the relationship between prevalence and virulence could thus be modified by integrating the other correlates of high prevalence.

The same type of frequency-dependent costs have also been found in the evaluation of inbreeding depression for several plant species [23–25]: The depression is generally greater when the competitors of an inbred individual are mostly outbred, as opposed to when they are mostly inbred. The existence of frequency-dependent costs for these two very different biological phenomena suggests that indirect costs due to the modification of intraspecific competitiveness could be common. Taking the composition of the population into account when evaluating a cost could thus be worthwhile for other phenomena.

### Evolutionary Implications

Modifications of the competitive ability of hosts by parasites have been shown to affect the population dynamics of hosts and parasites [17,18]. For example, in our system, in low-prevalence conditions, the strength of intraspecific competition against infected hosts is intense and leads to a large increase in developmental time. This enhances the parasite's potential transmission success and may help it to become established in naïve host populations by reducing the invasion threshold.

However, the evolutionary consequences of the modification of host competitiveness by parasites have not been considered, and in particular, models of virulence evolution [19,26–30] have not included it.

Prevalence-dependent virulence may also influence the evolution of constitutive host resistance to parasites. Indeed, in models for the evolution of resistance to parasites [31], low prevalence represents conditions in which the cost of resistance greatly reduces the fitness of resistant individuals, because a majority of them pay the cost without receiving the benefit of carrying a resistance allele. Low prevalence is thus a condition in which resistance to a parasite is counterselected. A negative relation between prevalence and virulence, as suggested by our results, would increase the relative fitness advantage of resistant individuals in populations with low prevalences of infection and could partially counterbalance selection against resistant individuals. This verbal model needs to be substantiated by a theoretical approach that includes a more realistic view of infection characteristics such as environmental and temporal variation in prevalence, parasite burden, or multiple infections.

In conclusion, our experiment reveals that, in our system, the outcome of competition among host larvae not only depends on the infection status of the individual concerned but also on that of its competitors. If this result is true in other host-parasite systems, it has several possible consequences: From an experimental point of view, the measurement of the parasite's virulence can be affected by the prevalence under which the measure is made. At the population scale, the virulence of the parasite varies with its prevalence, and this may affect the dynamics of the host-parasite system. From an evolutionary point of view, this phenomenon may influence the evolution of parasite virulence and host resistance.

## Materials and Methods

### Biological system

The microsporidium *V. culicis* naturally infects several genera of mosquitoes [32]. Host larvae ingest the parasites' spores along with their food and become horizontally infected when these spores germinate and infect host gut cells. Within host cells, *V. culicis* undergoes a series of developmental stages before starting to produce its spores from 8 to 10 days postinfection. Physical damage to host tissues occurs when spore-laden cells rupture and disseminate their contents. As the parasites' spores do not resist desiccation [33], its transmission success is more likely to be assured by spores released from the body of dead and decaying larvae or pupae rather than from infected individuals that emerge and leave the aquatic environment as adult mosquitoes (p. 455, [34]). Thus, larval developmental time and survival to adulthood are important life-history traits influencing the parasite's transmission success.

*Ae. aegypti* is a subtropical mosquito whose larvae grow in natural or artificial containers [35]. These sites show temporal variation in size and food availability, so larvae experience variable conditions of intraspecific competition. Larvae feed by filtering water, while pupae do not feed.

In previous studies, we found that intraspecific competition among uninfected *Ae. aegypti* larvae can strongly influence their developmental time and survival to adulthood [36,37]. Intraspecific competition not only involves competition for food [38], but includes all the detrimental environmental changes induced by the presence of conspecifics. These modifications can be stress-generated by physical contacts [39] or chemical pollution (e.g., by nitrogenous waste [40]), as previously demonstrated for *Ae. aegypti* larvae [41]. Concerning the effects of parasitism, we have shown that *V. culicis* prolongs developmental time and reduces the chances of reaching adulthood for *Ae. aegypti* larvae reared in the absence of intraspecific competition [42]. Furthermore, the expression of the parasite's virulence varied along a gradient of environmental resource availability, reflecting the strength of interspecific competition between host and parasite for host resources [42]. These results suggest that intraspecific competition is likely to interact and influence the expression of the parasite's virulence.

### Experimental design

Our strain of *Ae. aegypti* is derived from a large number of eggs collected in Tingua, Brazil and provided by Ricardo Lourenço de Oliveira of the Instituto Oswaldo Cruz (Rio de Janeiro, Brazil). It had been reared in standardised and outbred conditions (3,000 reproductive adults in each generation) in our laboratory for three generations at the time of the experiment. The spores of *V. culicis* were derived from a stock isolated from *Ae. albopictus* in Florida and provided by Dr. J. J. Becnel (United States Department of Agriculture, Gainesville, Florida, United States).

Recently hatched *Ae. aegypti* were split into 30 groups of 60 larvae each, and each group was put in a petri dish (diameter 55 mm) containing 10 ml of softened water. We added 6 × 10^4^ *V. culicis* spores per larva in 0.05 ml of softened water to 15 of the dishes. These latter individuals are subsequently called “exposed individuals.” To the remaining 15 petri dishes, we added 0.05 ml of softened water; we refer to these as “control individuals.” To each dish, we added 3.6 mg of Tetramin (powdered fish food). Spores and larvae were kept in contact for 24 h. Contact between larvae and spores was restricted to 24 h in order to synchronise the age structure of infections.

After this infection period, larvae were rinsed and transferred to individual *Drosophila* vials (diameter 25 mm × 95 mm), two per vial, containing 5 ml of softened water.

We had three categories of vials, representing the three cases of intraspecific competitive interactions in a partially infected host population: (i) Vials containing two control larvae, (ii) vials containing two exposed larvae, and (iii) vials containing a control larva and an exposed larva. This minimalistic approach of manipulating density has already been shown to capture the general effects of intraspecific competition, while avoiding pitfalls of more traditional approaches [37]. During their development, larvae were provided daily with their food dissolved in 1 ml of softened water. Prior to feeding, 1 ml of water was removed to maintain a constant volume, because the depth of the environment in which larvae grow has been shown to influence mosquito life history traits [43].

Two food regimes were adopted: 50% and 100% of a standard regime. The standard regime consisted of 0.08 mg on day 1, 0.16 mg on day 2, 0.32 mg on day 3, and 0.64 mg of Tetramin per vial from day 4 onwards. In the 50% food regime, these quantities were divided by two. Food was provided to vials until both individuals in each vial had either died or pupated, and the amount of food was not adjusted to the number of larvae remaining in the vial. This procedure was followed to allow the maximum number of individuals to reach adulthood and their gender to be determined, thus maximizing statistical power in the analysis of gender effects. Vials were arranged in racks of four-by-ten vials where each rack was assigned to a food treatment, half receiving the 50% regime and half the 100% food regime. The three infection treatments were distributed randomly among racks. The number of vials in each category was calculated, allowing for a 95% infection rate and sex-ratio variation, so as to obtain a minimum of 30 replicates in each infection/food/sex combination. There was a total of 775 vials. The experiment was divided into seven blocks in order to reduce the effects of uncontrolled environmental variations. The experiment was conducted in a room maintained at 25 °C and a photoperiod of 12 h of light to 12 h of dark.

Vials were examined every 12 h, and age at pupation was recorded. Pupae were transferred to individual vials containing 5 ml of softened water and the vials were covered with a fine nylon gauze. At emergence, the adult sex was noted. For all individuals (larvae, pupae, and adults) having an age at death of more than 8 d and coming from “+/+” and “+/−” treatments and 89 individuals coming from the “−/−” treatment, spore load was evaluated by homogenising the body of the individual in 0.2 ml of water and counting the number of spores with a Neubauer cell counter and phase-contrast light microscope. We considered as infected all individuals for which we observed more than one spore, each spore observed under the microscope corresponding to 2,000 spores within an infected mosquito. Subsequent analyses were restricted to vials whose a posteriori infection status matched the a priori status. Dead mosquitoes were stored at −20 °C.

### Statistical analyses

The probability of emerging as an adult in the “+/+” and “−/−” treatments was estimated by comparing the frequency of vials in which both individuals emerged as adults as opposed to vials in which at least one individual died before this stage. From these data we estimated the probability of an infected or uninfected individual emerging. These estimates were used to generate predictions for the frequency of emergence in the “+/−” treatments and against which the observed frequencies were tested. Only vials we could confirm as not having their a priori infection status were excluded from these analyses, those which could not be confirmed because of mortality before spore production began were assumed to have their a priori infection status.

Only replicates in which both individuals survived to pupation were used for the subsequent analyses. This insured that larval mortality did not influence traits of the surviving individuals. Moreover, as mentioned above, we included in the analyses only individuals from the vials in which the observed infection status matched the expected status. The size of the dataset was thus reduced to 539 vials.

The analysis presented here was specifically designed to answer the question, Do infected hosts exert more or less intense intraspecific competition than uninfected hosts? In a previous study [36], it was shown that the sex of a competitor could influence the intensity of competition. The sex of the competitor was thus included in our analyses. One of the two mosquitoes in each tube was randomly selected as the focal individual to be analysed, with the other designated as its competitor. We combined our knowledge of the infection status and sex of both individuals into two factors, infection status and sex status. There were four categories in each factor. For infection status, these categories were (i) infected versus infected (+/+), (ii) infected versus control (+/−), (iii) control versus infected (−/+), and (iv) control versus control (−/−), where the first term refers to the focal individual and the second to its competitor. Correspondingly, the categories for sex status were (i) female versus female, (ii) female versus male, (iii) male versus female, and (iv) male versus male, with first and second terms referring to the focal and competitor individuals, respectively. As it is only data from the focal individual being analysed, the degrees of freedom in our analysis are based on the number of replicate vials and not on the number of individuals in the data set.

The factors of infection status, sex status, and food regime were analysed by fully factorial ANOVA. However, the results of such an analysis may have depended on the particular combination of mosquitoes randomly selected as the focal individual. Consequently, we resampled the data by repeating the randomisation process and analysis 200 times. We had previously verified that the mean of the estimated F-values was stable with fewer than 200 resamplings. The results presented correspond to the mean of estimated values obtained by the resampling procedure. The *p*-values presented are from the upper 95% quantile of the distribution of *p*-values obtained and are, thus, conservative estimates. Based on the results of this first analysis, we decided to test for the effect of the nature of the competitor. To do so, we first performed the same type of analysis (with resampling and ANOVA) on a data file containing only “+/+” and “+/−” individuals to test the effect of the nature of the competitor on infected individuals. We then performed the same type of analysis on a data file containing only “−/+” and “−/−” individuals to test for the effect of the nature of the competitor on control individuals. These analyses had sex status, infection status, and food regime as factors, and are equivalent to a contrasts analysis.

Statistical analyses were performed with JMP, version 3.2.2 (SAS Institute, http://www.sas.com/) and Splus 2000 (MathSoft, http://www.mathsoft.com/).

### Life history traits

We analysed the effects of competition and parasitism on the probability of emergence and developmental time. More specifically, we did not examine their effects on adult body size or starved adult longevity because of the food distribution pattern we followed in this experiment. Indeed, as mentioned above, a given amount of food was distributed to each vial daily until both individuals of the vial had either pupated or died, implying that after the first individual had pupated the second received the assigned amount of food for two individuals. Therefore, slow-developing individuals received more food than fast-developing individuals, which renders body size and longevity data uninterpretable. The food provisioning bias can only weaken the effects we reveal on survival and developmental time, which renders our analyses on these traits conservative in this respect.

## Abbreviation

d.f.: degree(s) of freedom
